# Universal switching of plasmonic signals using optical resonator modes

**DOI:** 10.1038/lsa.2016.237

**Published:** 2017-06-02

**Authors:** Cillian PT McPolin, Nicolas Olivier, Jean-Sebastien Bouillard, Daniel O'Connor, Alexey V Krasavin, Wayne Dickson, Gregory A Wurtz, Anatoly V Zayats

**Affiliations:** 1 Department of Physics, King’s College London, Strand, London WC2R 2LS, UK; 2 Present address: University of Sheffield, Hounsfield Road, Sheffield S3 7RH, UK; 3 Present address: University of Hull, Cottingham Road, Hull HU6 7RX, UK; 4 Present address: National Physical Laboratory, Hampton Road, Teddington, Middlesex TW11 0LW, UK; 5 Present address: University of North Florida, 1 UNF Drive, Jacksonville, FL 32224, USA

**Keywords:** Fano resonances, optical signal processing, plasmonics, ultrafast switching

## Abstract

We propose and investigate, both experimentally and theoretically, a novel mechanism for switching and modulating plasmonic signals based on a Fano interference process, which arises from the coupling between a narrow-band optical Fabry–Pérot cavity and a surface plasmon polariton (SPP) source. The SPP wave emitted from the cavity is actively modulated in the vicinity of the cavity resonances by altering the cavity Q-factor and/or resonant frequencies. We experimentally demonstrate dynamic SPP modulation both by mechanical control of the cavity length and all-optically by harnessing the ultrafast nonlinearity of the Au mirrors that form the cavity. An electro-optical modulation scheme is also proposed and numerically illustrated. Dynamic operation of the switch via mechanical means yields a modulation in the SPP coupling efficiency of ~80%, while the all-optical control provides an ultrafast modulation with an efficiency of 30% at a rate of ~0.6 THz. The experimental observations are supported by both analytical and numerical calculations of the mechanical, all-optical and electro-optical modulation methods.

## Introduction

The implementation of energy-efficient and ultrafast functionalities for the generation and control of electromagnetic radiation in nanoscale devices represents a prerequisite for the development of ultra-integrated photonic circuits. Plasmonic components, together with hybrid plasmonic–dielectric structures, enable strong light–matter interaction, thereby allowing the realization of small-footprint, low-power and high-efficiency switches and modulators of optical signals in an integrated manner^1^. Such components have been achieved by incorporating electro-optic or nonlinear materials^2^, quantum dots and photochromic molecules into plasmonic structures^3, 4^ to increase weak refractive or absorptive changes of the functional media. The modulation speed and depth are both critical factors for many applications, including data transport and processing, and consequently efficient ultrafast control is currently the focus of considerable research efforts. Furthermore, minimizing the switching energy is essential for the on-chip integration of plasmonic devices and ensuring a broad range of practical applications.

A promising route for achieving this goal involves employing nonlinear plasmonic structures, which provide the opportunity to manipulate optical and plasmonic signals with low switching energies, due to large resonant local fields^5^. Moreover, plasmonic excitations may respond on a short timescale, typically a few tens of femtoseconds to picoseconds, presenting an avenue for ultrafast electromagnetic signal control at the nanoscale. Using the nonlinearity of plasmonic metals and adjacent dielectrics, several approaches to switching and modulation have been investigated, including controlling the dispersion of Bloch modes in plasmonic crystals^6, 7^, manipulating the coupling efficiency to surface plasmon polariton (SPP) modes^8^ and optically modulating plasmonic losses of an aluminum SPP waveguide^9^. In addition to planar structures, plasmonic metamaterials have been shown to facilitate ultrafast control of photonic signals. These modulation processes can be tailored to, and enhanced at, specific wavelengths^10, 11^, providing nonlinearity that can be extensively engineered through geometrical means^12^. Plasmonic metamaterials, nano-antennas and components can also be integrated into dielectric or Si-photonic circuitry to achieve active functionalities^13, 14, 15, 16^.

In this paper, we propose a novel SPP switching scheme that is efficient under various control methods, including mechanical, electro-optical and all-optical operation, whilst remaining compatible with other existing photonic and plasmonic components. Specifically, in the all-optical implementation, the switch provides ultrafast modulation speeds with practically useful modulation depths, together with the potential for low switching energy. In addition, high modulation depths are easily achieved using mechanical control. The underlying mechanism of the switch is based on the coherent coupling between a spectrally sharp Fabry–Pérot (FP) cavity resonance and a broadband SPP source, with the relative phase relationship between the two providing an efficient means to control the emitted SPP power. Moreover, the switching process may be viewed in terms of interfering oscillators and thus understood as a Fano effect. The sensitivity of Fano resonances is particularly advantageous for switching and sensing applications^17, 18, 19, 20, 21, 22, 23^.

## Materials and methods

### Sample fabrication

**Figure 1:**
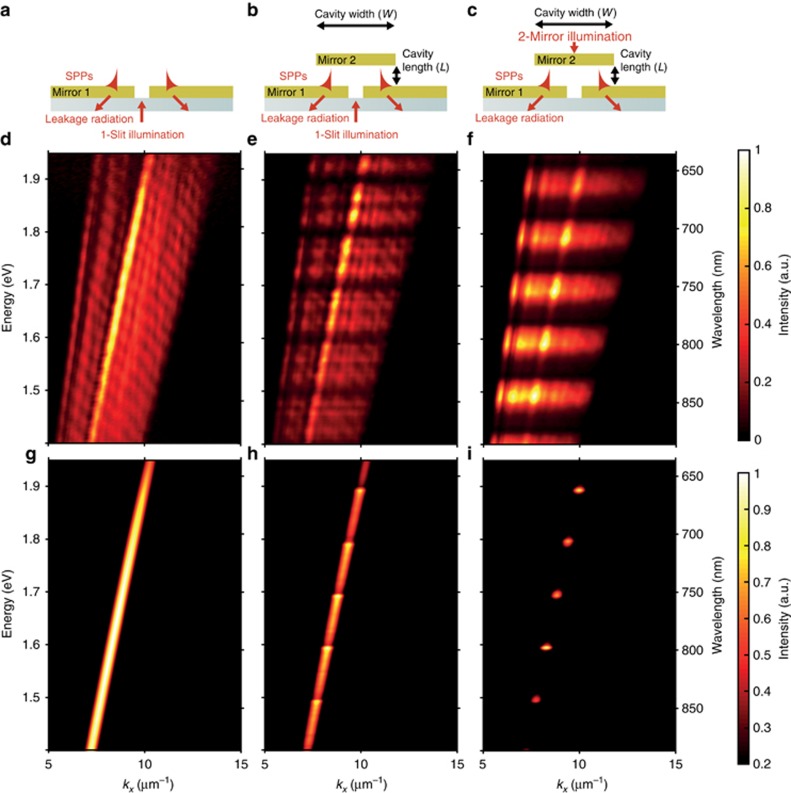
(**a**) SPP excitation with a slit in a metal film. (**b**, **c**) Geometry of the switch composed of a cavity formed by two metal films with the bottom one (mirror 1) containing a slit and also acting as an SPP waveguide. The two films are separated by an air gap *L* defining the cavity length. The top film (mirror 2) has a finite width *W*. The cavity can be illuminated through the slit (**b**) or the top mirror (**c**). (**d**–**f**) Experimentally measured and (**g**–**i**) simulated dispersions of SPPs under the excitation conditions (**a**–**c**), respectively. The thickness of mirrors 1 and 2 is 50 nm and the slit width is 150 nm. The width *W* of mirror 2 is on the order of 10s of microns. The cavity length *L* is approximately 6 μm. The contrast in each dispersion (**d**–**i**) is normalized to the maximum intensity. The broader resonances in **f** compared with **e** may be due to different collimation of illuminating light.

Using a focused ion-beam microscope, a slit was milled in a 50 nm-thick gold (Au) film that also serves as the supporting medium for the SPP modes that are excited upon illumination. The slit width of ~150 nm provides a broad transmission spectrum and significant wavevector spread of scattered light, ensuring SPP excitation in the visible region. The FP cavity is formed between two 50 nm-thick mirrors (Figure 1b). This geometry imposes a significant cavity length, on the order of microns, ensuring that the interaction between the two films is minimized for SPPs leaking into the substrate. The upper mirror was formed by thermally evaporating a 50 nm gold film onto a glass slide that was subsequently attached to a three-dimensional-printed custom holder. This in turn was mounted onto a piezo stage, allowing *x*–*y*–*z* control. The design of the three-dimensional-printed holder ensured that the upper mirror was positioned relatively parallel with the lower gold film. In addition, the presence of air inside the cavity, instead of a dielectric material, minimizes undesirable intra-cavity SPP refraction and interference as the plasmonic signal transmits from the resonator, of finite lateral width, to the isolated Au film. It is also important to emphasize that air was chosen to fill the cavity to guarantee the emergence of leakage radiation in the substrate, originating from SPPs, which is the observed experimental quantity. An effective switch could otherwise take various geometries, including a cavity composed of two extended films in a homogeneous dielectric environment.

### SPP coupling efficiency measurements

Supplementary Figure S1 shows the experimental setup used to measure the SPP intensity, via monitoring the leakage radiation intensity, and to demonstrate dynamic signal modulation. Incident radiation scatters from the slit, thus exciting SPPs that subsequently tunnel through the thin Au film and couple to light propagating in the substrate. An oil immersion objective (100 ×, numerical aperture (NA)=1.42) was used to collect this leakage radiation that was then imaged in Fourier space by the 4-lens arrangement, yielding the SPP dispersion when the structure is illuminated by a white light beam^24^. The leakage radiation intensity is directly proportional to the experimental coupling efficiency, with this coupling efficiency normalized to the maximum measured value. Removing lens L3 allowed the real space to be imaged, whilst real and Fourier space filters may be placed in the corresponding planes.

### Mechanical modulation

The position of the top mirror in Figure 1b and 1c is controlled by a closed-loop piezoelectric stage, which also permits the cavity length to be mechanically varied. No active feedback was employed in the mechanical modulation experiment, but rather the piezo stage was continuously raster scanned in the vertical direction for improved stability. The cavity length was then determined from the measured mode spacing, via the equation *L*=*πc*/Δ*ω*_*r*_, where *L* is the cavity length and Δ*ω*_*r*_ is the mode spacing. The illuminating white light can be fed into the cavity either via the slit or through the top mirror (Figure 1).

### Optical modulation

Transient pump-probe measurements were performed with a 200 fs transform-limited, 460 nm wavelength pump pulse, which controls the state of the switch while a time-delayed chirped 1 ps broadband white light super continuum probe pulse acts as the signal. A delay line controls the temporal delay between the pump and probe pulses, providing a time step on the order of 50 fs. Scanning the delay line thus allowed the modulation dynamics to be recovered. The measurements were performed in the slit illumination conditions described in Figure 1b. A beam block, positioned in an intermediate Fourier plane in the collection system, filters out the zero-order reflection from both the signal and pump pulses, whereas stop-band filters remove the remaining scattered pump intensity (Supplementary Fig. S1). In the measurements, a time delay *τ*=−1.1 ps corresponds to the ground-state response of the cavity system, the same as *τ*→∞.

### Numerical modeling

The studied device was numerically modeled using commercial finite-element method software (COMSOL Multiphysics 4.3a) applying gold permittivities taken from Ref. 25. A monochromatic Gaussian beam (full width at half maximum=6 μm) was used to illuminate the structure at normal incidence, and the SPP intensity was monitored by performing a fast Fourier transform on the magnetic field. The nonlinear optical dynamics were modeled using the two-temperature model^12^ (Supplementary Note 1). The voltage-induced changes in indium tin oxide (ITO) permittivity were modeled according to the method described in Ref. 16.

## Results and discussion

We consider a generic geometry consisting of a plasmonic waveguide with an optical cavity formed by a mirror positioned at some distance from the waveguide, with both layers parallel with respect to each other (Figure 1b and 1c). To utilize the cavity as a modulating element for SPP signals, the illuminating light can be delivered to the cavity either through a slit, which acts as the SPP source, placed in the waveguide (Figure 1b) or through the semi-transparent mirror (Figure 1c). Illuminating the cavity through the slit results in the transmitted intensity feeding the cavity and partly coupling to SPPs via scattering by the slit. This direct coupling pathway to SPPs is typically not very efficient when considering the waveguide alone. For example, the numerically modeled SPP coupling efficiency is ~10% at a free-space wavelength of 850 nm for a 100 nm wide slit in a 200 nm-thick Au film on a glass substrate. This efficiency is estimated as the ratio between the power in the SPP mode 10 μm away from the slit, taking into account SPP propagation loss, to the power incident on the slit aperture (Supplementary Note 2).

In contrast, the cavity geometry allows light not coupled to SPPs upon the initial slit diffraction to be reflected back towards the slit by the upper mirror, providing another opportunity for scattering and, thus, SPP excitation. If the light frequency is close to the FP resonance frequency, multiple reflections within the cavity can provide in-phase enhancement of the light emitted by the slit, yielding an increased light-to-SPP coupling efficiency of tens of percents compared with either the metal film without a cavity or in off-resonance conditions^26^. We may consider this to be an indirect pathway for SPP excitation, as it relies on light that initially scattered from the slit without coupling to SPPs. The efficiency of this indirect pathway to excite SPPs, originating from light trapped within the cavity, depends on the resonant properties of the cavity, including the resonant frequency and Q-factor, and can therefore be controlled by altering the modal properties of the cavity.

This effect paves the way for efficient switching and modulation of plasmonic signals. As optical cavity modes are naturally very sensitive to any changes in the path length (shifting the resonances) or losses (altering the Q-factors of the resonances), efficient switching can be designed for any given wavelength by choosing the appropriate geometry of the cavity and refractive index of a material inside it. Thus, the proposed approach provides a universal geometry for the modulation of plasmonic signals, offering dynamic control through different means, including micromechanical motion of the mirror, all-optically altering the resonance conditions via third-order Kerr optical nonlinearities, through electro-optical effects, and other effects that influence the cavity modes.

### SPP excitation efficiency in the presence of cavity resonances

Figure 1d shows the SPP dispersion measured for an Au–air interface without the presence of the top mirror (Figure 1a), obtained by exciting SPPs with white light transmitted through the slit. When the top mirror is positioned above the slit, forming a cavity with a length of ~6 μm (Figure 1b), a series of dark bands appear in the dispersion (Figure 1e), indicating a reduction in the SPP excitation efficiency at the frequencies of the cavity resonances. The reduction in leakage radiation extends beyond the SPP dispersion range, along the cavity resonances, clearly indicating a modification of the slit emission properties when coupled to the modes from the cavity. The resonant frequencies for such a cavity occur at *ω*_*r*_=*rπc*/*L*, where *r* is an integer corresponding to the mode number, *L* is the cavity length and *c* is the speed of light. The SPP dispersion in Figure 1e contains five dispersion-less (equidistant by ~0.1 eV) cavity modes in the frequency range *ħω*_*r*_=1.45–1.95 eV, corresponding to *r*=15–19. When illuminating the cavity with white light through the top mirror instead of through the slit (Figure 1c), the same five resonances are observed in the dispersion. However, they now appear as bright bands instead of intensity minima, indicating an increase in the SPP coupling efficiency (Figure 1f).

These observations can be understood by considering the interference between the broad continuum of the slit diffraction and the narrow resonances supported by the cavity. The frequency dependence of this Fano-type interference can be expressed as 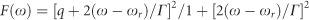, where *Γ* is the resonance linewidth, and the Fano parameter *q* describes the ratio between indirect, resonant scattering and direct, non-resonant scattering^26, 27, 28, 29, 30^.

**Figure 2:**
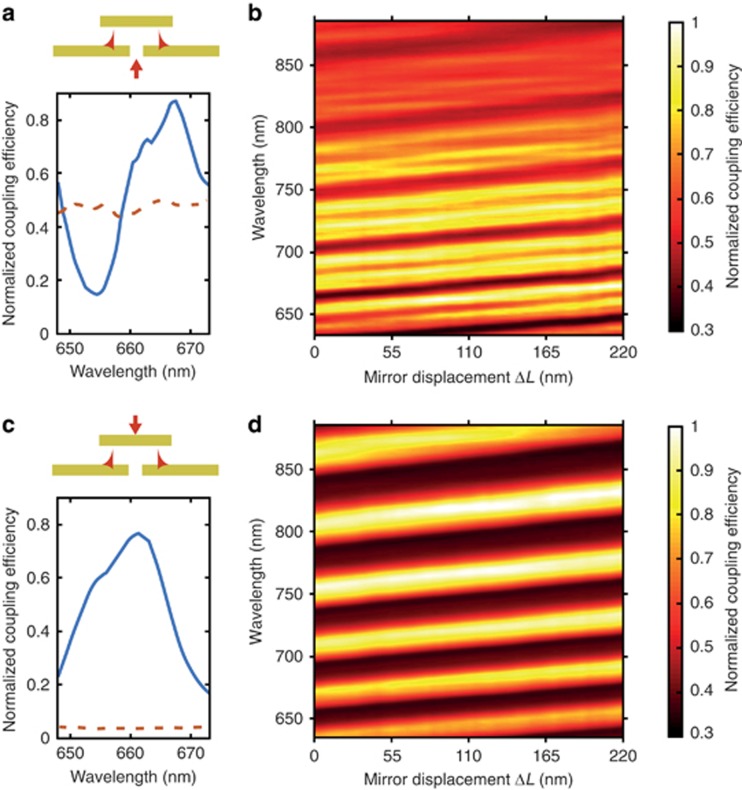
(**a**, **c**) The spectral dependences of the coupling efficiency obtained under (**a**) slit- and (**c**) mirror-illumination conditions plotted from the cross-section of **b** and **d**, respectively, for (solid lines) a fixed cavity length *L* and (dashed lines) a single film without the cavity. Inserts in **a** and **c** illustrate the illumination geometry. (**b**, **d**) SPP coupling efficiency spectra as the cavity length increased by Δ*L* for (**b**) slit- and (**d**) mirror-illumination conditions. The coupling efficiencies are normalized to their maximum values. Other parameters are as in Figure 1. **b** corresponds to a cross-section along the SPP dispersion of Supplementary Movie S1.

**Figure 3:**
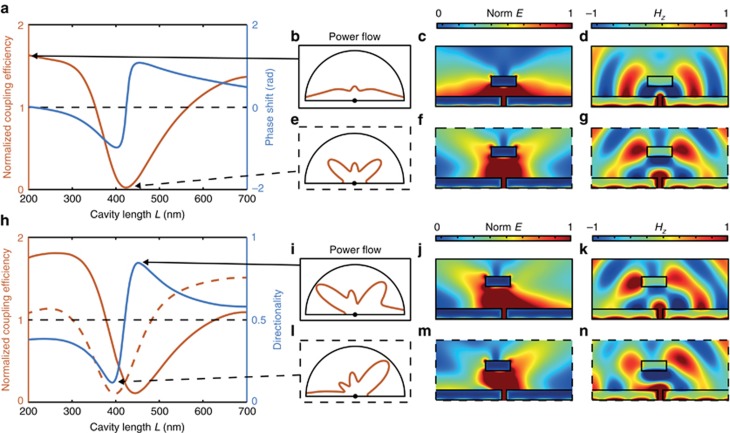
(**a**) Simulated dependence of the SPP coupling efficiency on the cavity length *L*. The SPP coupling efficiencies in **a** and **h** have been normalized to the cavity-less case (that is, without mirror 2) and, thus, a value greater than unity represents enhanced coupling. (**b**, **e**) Angular emission diagrams, calculated from the power flowing through a 1.5 μm radius hemisphere centered on the slit opening, for (**b**) off and (**e**) on FP resonance conditions. (**c**, **d**, **f**, **g**) Field distributions in and around the cavity for (**c**, **d**) off- and (**f**, **g**) on-resonance conditions. (**h**) As **a** but in the case of the asymmetric cavity with the upper mirror displaced to the left by 125 nm for the SPPs propagating to (dashed lines) right and (solid lines) left. Directionality is defined as *D*=*P*_*r*_/(*P*_*r*_+*P*_*l*_), where *P*_*r*_ and *P*_*l*_ are the SPP power flows in the right and left directions, respectively. (**i**, **l**) Angular emission diagrams at the maximum (**i**) right and (**l**) left SPP coupling efficiencies. (**j**, **k**, **m**, **n**) Field distributions in and around the cavity for the SPP excitation to the (**j**, **k**) right and (**m**, **n**) left. The simulations have been performed for a cavity width *W*=500 nm and an incident light wavelength of 850 nm, with the gold films both 200 nm in thickness and a slit width of 100 nm. **c**, **f**, **j** and **m** are the norm of the electric field and **d**, **g**, **k** and **n** are the out of plane magnetic field. The center of the slit aperture in the emission diagrams is indicated by a black point in **b**, **e**, **i** and **l**.

Illumination of the cavity through the slit leads to asymmetric lineshapes in the SPP dispersion at frequencies in the vicinity of the cavity resonances (Figures 1e, 2a and 2b). In this case, the Fano regime is characterized by the parameter *q* being close to unity (*q*~1), as the direct and indirect scattering processes have similar amplitudes. The resulting SPP intensity exhibits a considerable spectral modulation with both a twofold enhancement and suppression compared with the single film geometry (Figure 2a). There is a general decrease in SPP coupling efficiency at higher wavelengths (Figure 2b), which is due to the wavelength-dependent scattering efficiency from the slit, and is not related to the presence of the cavity. The observed modulation arises from the modification by the FP cavity of the emission properties of the slit, both in term of emitted power and radiation diagram (Figure 3b and 3e), clearly showing that the modulation in the SPP excitation efficiency follows a change in the source in-plane scattering intensity. Moreover, considering the simulations of Figure 3, whereas the total power emitted by the slit is only reduced by ~8% going from an off-resonance (*L*=200 nm) to on-resonance (*L*=425 nm) cavity excitation, the SPP power changes by almost 99%, an effect thus mainly governed by a change in the slit emission diagram.

When the cavity is fed through the top mirror, the SPP resonance lineshapes are symmetric, illustrating only resonant enhancement (Figures 1f, 2c and 2d), which is indicative of the enhanced field at the cavity mode frequencies scattered by the slit edges into SPP modes. This regime corresponds to a Fano parameter *q* much greater than unity (*q*>>1), as indirect resonant scattering becomes the dominant process.

The observed change in SPP intensity near the cavity resonances equates to a modification of the SPP coupling efficiency. In addition, SPPs may be excited on the upper mirror due to scattering from the mirror edges, provided the mirror width *W* is comparable to the beam width, or roughness of the Au films. However, in the experiment, the beam is much smaller than the mirror width and thus SPP excitation is negligible. Moreover, the separation between the two films is on the order of microns and hence any SPPs on the upper mirror would not significantly interact with those on the lower one. Numerical simulations for the geometries equivalent to the experiment exhibit excellent agreement with the observed dispersions (Figure 1g–1i). However, there is greater background light scattering in the experiments compared with the simulations, partially due to the roughness of the gold films. This is visible in Figure 1d–1f as broad bands surrounding the SPP dispersion curves. The black areas on either side of these bands are a consequence of filtering in the Fourier space and the finite NA of the objective.

The simulated electric field distributions when the cavity is driven on- and off-resonance also clearly show the change in SPP coupling efficiency (Figure 3c and 3f). The proposed switch has the potential to be scaled to sub-wavelength dimensions and provides the opportunity to control the directionality of SPP coupling by displacing the mirror laterally (Figure 3h–3n). In this asymmetric system, the resulting Fano resonances possess different lineshapes for the right- and left-propagating SPPs, hence illustrating the directional coupling at frequencies near the cavity modes. Altering the cavity length has the effect of changing directionality from ~0.2 to ~0.8 (Figure 3h), with the electric field profiles confirming the asymmetry.

### SPP switching through micromechanical modulation of the cavity length

We first consider the modulation scheme based on driving the cavity in and out of resonance, which was experimentally implemented by electro-mechanically controlling the position of the top mirror using a piezoelectric translator. This modifies the cavity length *L* and, as a result, shifts the cavity resonance frequencies by Δ*ω*_*r*_~(*rπc*/*L*^2^)Δ*L*. For example, a change in length by Δ*L*=200 nm corresponds to a change in the resonant frequency by Δ*ħω*_*r*_~0.06 eV for *r*=18, resulting in an observed 80% change in the SPP coupling efficiency in the slit-fed conditions (Figure 2b). This is equivalent to a modulation amplitude of 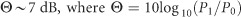, and *P*_1_ and *P*_0_ are the ‘on’- and ‘off’-state powers of the switch, respectively. Comparable modulation is observed at the wavelengths of higher cavity resonances (Supplementary Movie S1) and for the mirror feed geometry (Figure 2d). In addition to piezoelectric control, electrostatic and electrothermal schemes may also be implemented for this SPP modulation approach with high-frequency cantilevers or membranes acting as the second mirror, similar to those employed in the wavelength tuning of vertical-cavity surface-emitting lasers^31, 32^.

### Ultrafast SPP switching through optical modulation of cavity resonances

Ultrafast switching on the picosecond timescale can be achieved in all-optical schemes where a control beam is used to modulate the coupling of a probe beam to SPP modes, further illustrating the broad adaptability of the cavity-based system. To demonstrate all-optical modulation, the control light takes the same path as the signal light, both illuminating the cavity from the slit side. In this geometry, the modulation approach is to use the control light to modify the reflectivity of the cavity mirror, changing the Q-factor that governs the Fano process. A control beam at a wavelength of 460 nm and 200 fs in pulse duration primarily affects the bottom mirror’s reflectivity through a change in the imaginary part of the Au permittivity subsequent to optical absorption via both interband and intraband electronic excitation processes^10^. Although the real part of the Au permittivity is also modified in this process, the magnitude of the change, and therefore its effect, is much smaller, typically by about one order of magnitude. These changes in the permittivity of Au follow a control beam-induced rise in the electron energy, which is subsequently dissipated with a typical relaxation time on the order of a picosecond, mainly governed by electron–phonon scattering processes in Au. Hence, the nonlinearity of Au permits the Q-factor of the cavity to be adjusted on the picosecond timescale.

**Figure 4:**
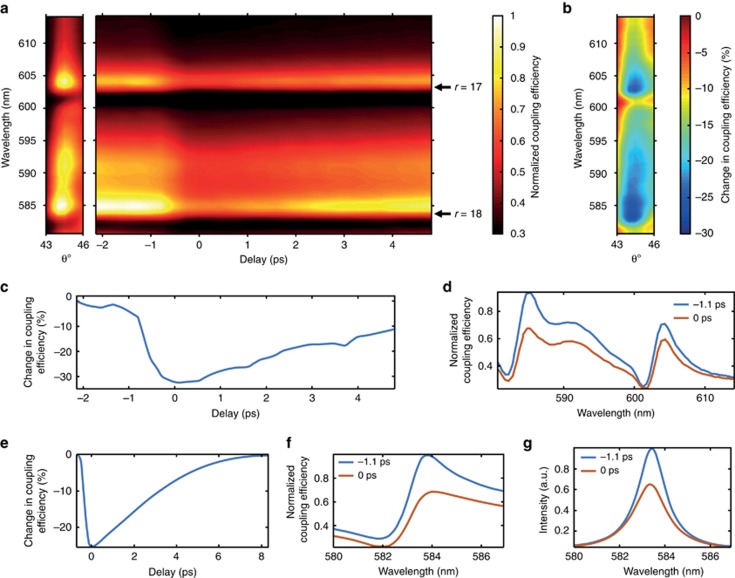
(**a**) Left: SPP dispersion measured under the slit illumination conditions (as in Figure 1e). Right: dynamics of the SPP coupling efficiency measured for different wavelength around the FP resonances *r*=17 and *r*=18 at *θ*=45°. (**b**) Differential SPP coupling efficiency for ground (*τ*=−1.1 ps) and excited (*τ*=0 ps) states plotted from **a**. (**c**, **e**) Dynamics of the differential coupling efficiency at a wavelength of 585 nm near the resonance *r*=18: (**c**) plotted from **a**, (**e**) simulations. (**d**, **f**) Spectra of SPP coupling efficiencies for ground (*τ*=−1.1 ps) and excited (τ=0 ps) states revealing the Fano resonances and their modification by the control pulse: (**d**) cross-sections from **a**, (**f**) simulations. (**g**) Simulated spectra of the direct transmission through both mirrors for ground (*τ*=−1.1 ps) and excited (τ=0 ps) states. The coupling efficiencies and intensities are normalized to their maximum values.

The dispersion of the excited SPP modes, measured from the leakage radiation for a small angular interval of interest (Figure 4a, left panel), reveals two minima in the SPP coupling efficiency, which are the result of the resonant excitation of the cavity modes at *λ*=602 and *λ*=584 nm for a cavity length of ~9 μm, again illustrating the distinct asymmetric Fano lineshape (Figure 4d). The time-dependent modification of the SPP dispersion, obtained for off-resonance control light with a center wavelength of 460 nm and a fluence of 0.45 mJ cm^−2^, is plotted in the right panel of Figure 4a around the arrival time of the control pulse, *τ*=0 ps, corresponding to the maximum temporal overlap between control and signal pulses. The cross-sections of these dependencies show that the general Fano lineshape is never lost throughout the transient process with the strongest effect coming from the attenuation of the peak at 585 nm, resulting in a change in the generated SPP intensity on the order of 30% (Figure 4b and 4c), corresponding to a modulation amplitude of approximately Θ∼1.5 dB. The slight modification in the resonance lineshape can be traced back to the changes in the ratio of direct to indirect scattering intensities. This affects the Fano parameter *q*, which decreases upon optical excitation due to an induced increase in cavity losses.

Numerical simulations of the experimental conditions confirm these considerations and also reproduce the coupling efficiency behavior (Figure 4f). The Q-factor was calculated to be Q~380 before the arrival of the control pulse (*τ*=−1 ps), decreasing to Q~300 when *τ*=0 ps, as can be seen in the transmission spectrum of the cavity (Figure 4g), which accounts for the variation in the Fano parameter. Moreover, the simulations show that this excitation energy corresponds to a peak electron temperature increase for the lower mirror to ~1580 K from the initial temperature of 300 K, subsequent to an absorption of ~65% of the incident energy, whereas for the upper mirror, the electron temperature reaches ~620 K upon absorption of ~10% of the incident control pulse energy. The resulting maximum change in the permittivity of Au of the lower mirror containing the slit is on the order of (Re(*ε*_Au_)_1580 K_−Re(*ε*_Au_)_300 K_)/Re(*ε*_Au_)_300 K_~3% and (Im(*ε*_Au_)_1580 K_−Im(*ε*_Au_)_300 K_)/Im(*ε*_Au_)_300 K_~125% in the same spectral range (580–620 nm), also confirming the dominant role of the imaginary part of the Au permittivity in the switching mechanism. The dynamics of the system, as determined experimentally from the transient dependence, is on the order of 1.5 ps (Figure 4a and 4c). This process is mainly governed by electron–phonon scattering in Au, as recovered through transient nonlinear simulations that retrieve the nonlinear dynamic response (cf. Figure 4c and 4e).

### SPP switching through electro-optical control of cavity resonances

**Figure 5:**
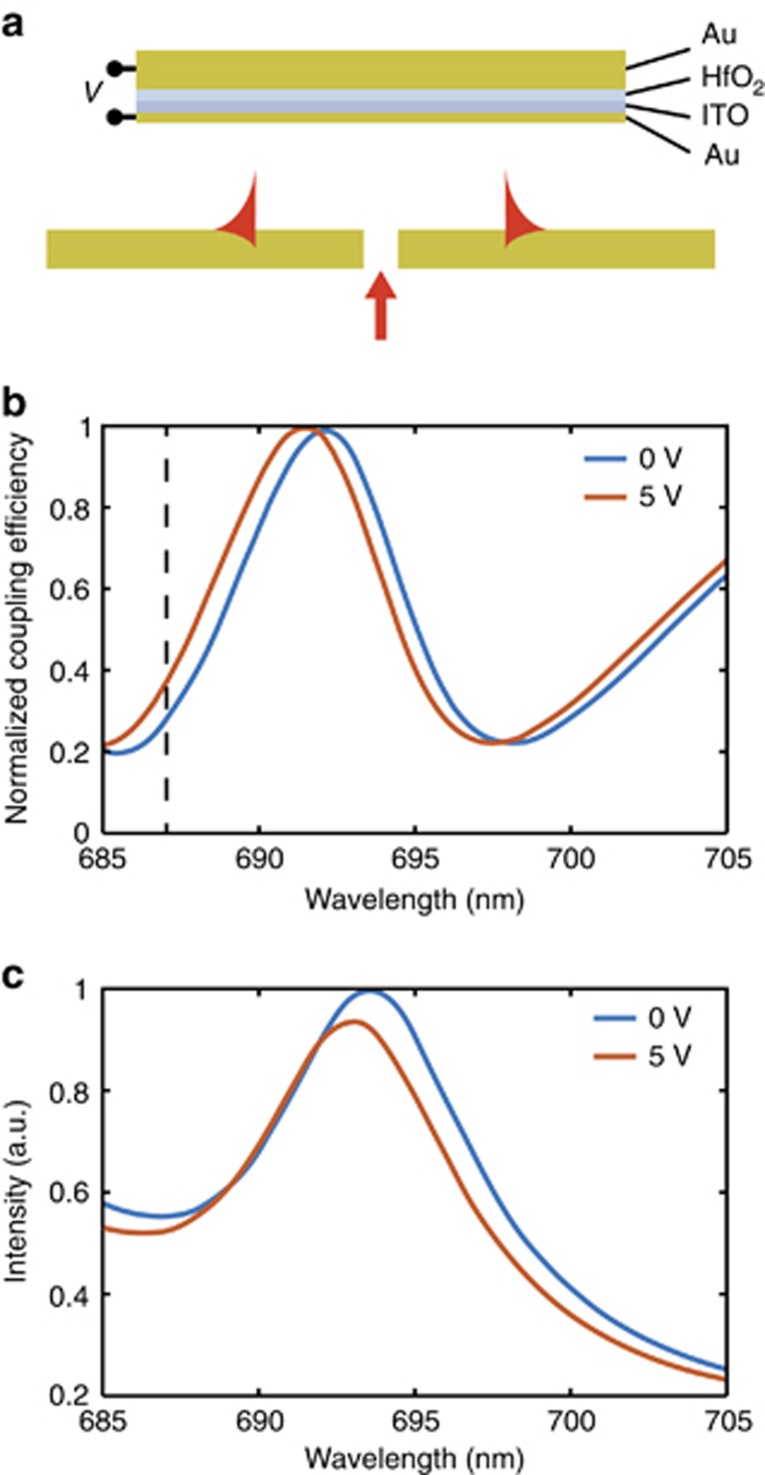
(**a**) Schematic of the electro-optical switching: the top mirror is the multilayer Au/ITO/HfO_2_/Au with the layer thicknesses of 50/2/2/2 nm, respectively. The 2 nm-thin Au layer acts as an electrode while also allowing significant optical transmission. Applying a voltage across multilayer mirror results in a change in reflection from the ITO–HfO_2_ interface due to charge accumulation. (**b**) The spectra of the SPP coupling efficiency with and without an applied voltage of 5 V. Dashed line at a wavelength of 687 nm indicates the spectral position of 30% change in coupling efficiency. (**c**) Transmission spectra of the cavity with and without an applied voltage of 5 V. The coupling efficiencies and intensities are normalized to their maximum values.

In addition to all-optical and mechanical modulation, the opportunity exists for electro-optical control of the switch. For example, this may be implemented via the refractive index modulation that occurs in ITO under the application of an electric field, which originates from an increased carrier concentration in the material^16, 33^. To employ this effect, without strongly affecting SPP modes at the Au/air interface, we numerically investigated a multilayer serving as the upper mirror, which consists of two gold films, acting as electrodes, separated by layers of ITO and HfO_2_ (Figure 5a). In this geometry, a voltage of 5 V applied between the electrodes leads to a change in the SPP coupling efficiency of up to 30%, which is equivalent to a modulation amplitude of approximately Θ∼ 1.5 dB. Although the SPP Fano resonance (Figure 5b) maintains the same general shape upon applying a voltage, it is blue-shifted, implying that the resonance frequency is modified. This is confirmed by the cavity transmission peak moving to a shorter wavelength (Figure 5c), due to the formation of an accumulation layer that effectively reduces the cavity length. Hence, whereas the cavity Q-factor changes from Q~110, without an applied voltage, to Q~100 when 5 V are applied, the SPP modulation primarily stems from a reduction in the effective cavity length.

## Conclusions

We have presented an effective switching configuration for plasmonic signals based upon a Fano interference process occurring between a broadband SPP source and a narrow-band cavity resonator. This interference process modifies the coupling efficiency of incident light-to-SPP modes. Choosing the cavity so that the illuminating light wavelength is near a FP resonance provides a universal means of efficiently controlling the SPP coupling efficiency by various schemes including micromechanical, nonlinear optical and electro-optical methods. In addition, the proposed switch offers the opportunity for controlling the directionality of SPP coupling, further expanding its functionality.

Experimental illustration of the dynamic modulation was achieved both mechanically, by spectrally shifting the cavity modes, and all-optically, by altering the Q-factor of the cavity resonances. The mechanical switch allows for a large change in the SPP coupling efficiency, exceeding 80%, whereas a two-color transient experiment demonstrated ultrafast SPP modulation with a change in the coupling efficiency of ~30% on the picosecond timescale. Effective implementation of mechanical control may be easily achieved via schemes similar to those employed in the wavelength tuning of vertical-cavity surface-emitting lasers. Moreover, to further improve the energy efficiency of this Fano-based switching device under nonlinear optical control, one can consider embedding nonlinear materials in the cavity, or altering the mirror material and geometry^34^, to augment the shift of the resonances. Such a configuration may also provide an opportunity to design multistable outputs of the switch.

The range of wavelengths at which modulation may take place is governed by the spectral position of the cavity modes. Increasing the cavity length would shift a given cavity mode to larger wavelengths and also increase the mode density in a given spectral range. In terms of exciting plasmonic modes, we have studied this using Au films in the visible to infrared spectral region. However, the same scheme would work at any wavelength, providing an appropriate plasmonic material was chosen (for example, Ag or Al in the ultraviolet spectral range) to support SPPs, or appropriate waveguides were implemented to support waveguided photonic modes. Moreover, the geometry described here provides a proof-of-principle demonstration, and may subsequently be optimized to yield improved performance. In principle, any geometry comprising a FP cavity coherently coupled to a source would provide the demonstrated switch functionality, and room for optimizing the performance of the switch is therefore likely.

In general, the proposed modulator geometry is amenable to interfacing with other plasmonic and photonic components, such as waveguides, detectors and sources, making it an ideal candidate for active nanophotonic applications, where integrated electro-optical, all-optical or micromechanical control of signals is required. The described general principle of the switching mechanism, which is based on the high sensitivity of the Fano resonances, can be easily extended to other functional geometries and operating wavelengths to create high-speed integrated devices for applications in biosensing, as well as in displacement, pressure and acoustic metrology.
