# Supplementary material for: Universal switching of plasmonic signals using optical resonator modes
Source: Light Sci Appl. 2017 Jun 2;6(6):e16237–. doi: 10.1038/lsa.2016.237 (PMC6062243; doi:10.1038/lsa.2016.237)
Supplement: Supplementary Information [file lsa2016237x2.docx]

**Supporting Information for**

**Universal Switching of Plasmonic Signals using Optical Resonator Modes**

Cillian P. T. McPolin^1,*^, Nicolas Olivier^1,2,*^, Jean-Sebastien Bouillard^1,3,*^, Daniel O’Connor^1,4^, Alexey V. Krasavin^1^, Wayne Dickson^1^, Gregory A. Wurtz^1,5,*^, and Anatoly V. Zayats^1,*^

^1^Department of Physics, King’s College London, Strand, London WC2R 2LS, UK

^2^Present address: University of Sheffield, Hounsfield Road, Sheffield S3 7RH, UK

^3^Present address: University of Hull, Cottingham Road, Hull HU6 7RX, UK

^4^Present address: National Physical Laboratory, Hampton Road, Teddington, Middlesex TW11 0LW, UK

^5^Present address: University of North Florida, 1 UNF Drive, Jacksonville, Florida 32224, USA

*These authors contributed equally to this work.

Correspondence: [cillian.mcpolin@kcl.ac.uk](mailto:cillian.mcpolin@kcl.ac.uk)


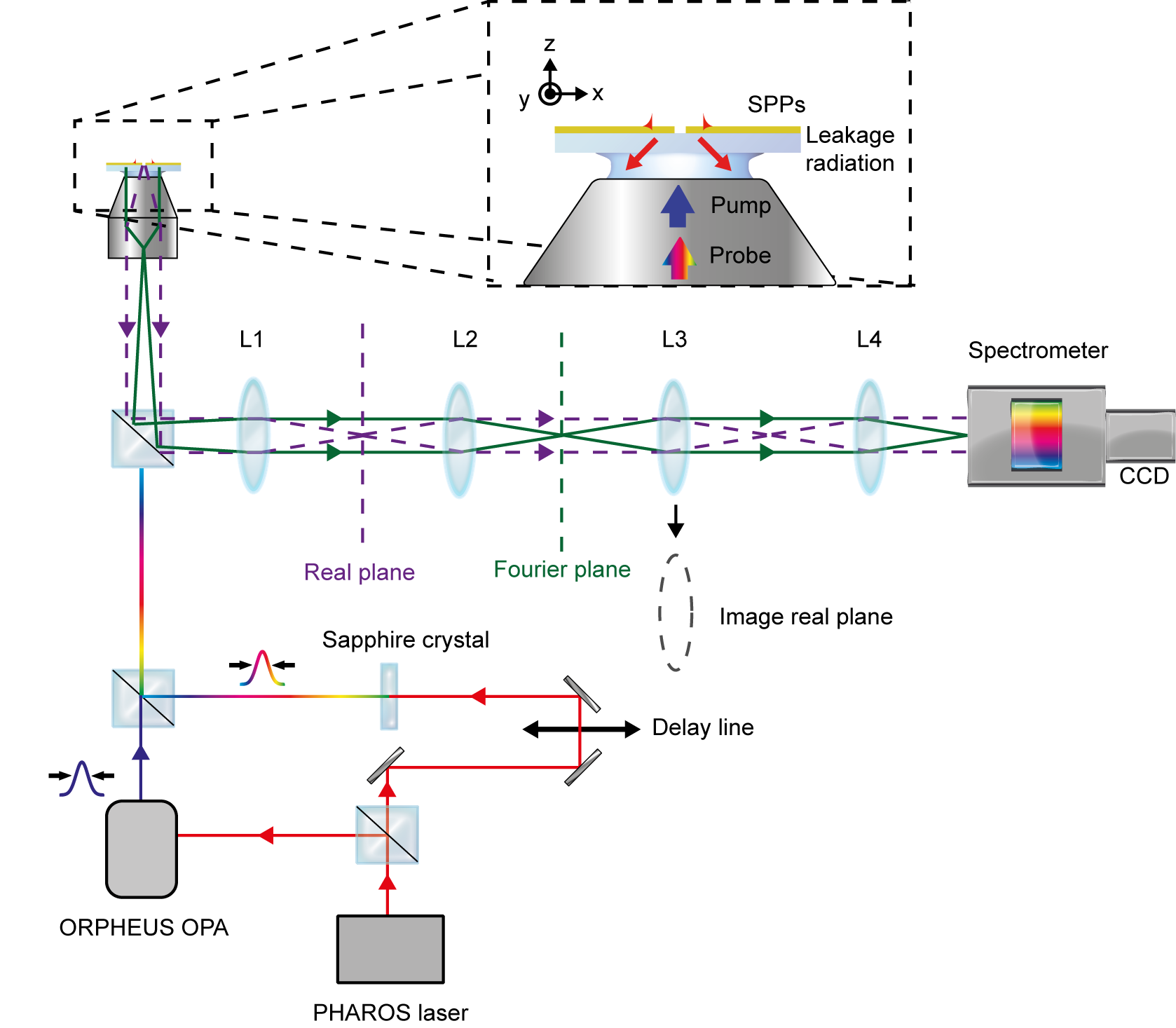


**Figure S1**. Experimental setup for the pump-probe measurements with Fourier imaging of the leakage radiation.

**Movie S1**. The experimentally measured variation of the SPP dispersion (a cross-section is shown in Figure 2b) as the upper mirror is moved up and down. The maximum mirror displacement is 220 nm. The cavity modes shift as the mirror is displaced, permitting micromechanical control over the SPP coupling. The structure parameters and experimental conditions are as in Figure 1e.

**Supplementary Note 1.**

**Modeling the Pump-Probe Experiments**.

Modeling the nonlinear Kerr-response of gold under pulsed optical excitation was done within the random phase approximation (RPA). In this context, the intensity-dependent permittivity of gold was expressed as the sum of both interband and intraband contributions:

$\varepsilon\left(\omega\right)=\varepsilon_{\text{inter}}\left(\omega\right)+\varepsilon_{\text{intra}}\left(\omega\right)$ (S1)

where the $\varepsilon_{\text{intra}}$ term relates to free electron motion within a parabolic sp band and $\varepsilon_{\text{inter}}$ relates to interband transitions between a dispersionless d band and the parabolic sp. The intraband contribution was expressed via a Drude-like response:

$\varepsilon_{\text{intra}}=\varepsilon_{\infty}-\frac{\omega_{p}^{2}}{\omega\left(\omega+i\gamma_{\text{intra}}\left(\omega\right)\right)}$ (S2)

where the plasma frequency is $\omega_{p}=2.168\times10^{15}\text{rad s}^{-1}$,
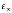
 is the high frequency permittivity, and$\gamma_{\text{intra}}\left(\omega\right)$ characterizes the damping rate of free-electrons. Moreover, the interband term was described within the RPA as:

(S3)

where $K=1.2695\times10^{32}$ is a wavevector independent constant related to the magnitude of the dipole transition moment for the interband transitions. $E_{g}=1.98\text{ eV}$ is the transition energy between the d and sp bands and $\gamma_{\text{inter}}$ is the scattering term for interband transitions. The term$f\left(x,T_{e}\right)$ is the Fermi-Dirac distribution for the electrons at an equilibrium temperature $T_{e}$.

Once a gold film is optically excited, the electrons respond almost instantaneously, and the energy subsequently decays via collisions amongst the electrons and lattice phonons, which possess equilibrium temperatures $T_{e}$ and $T_{L}$, respectively. The time response of the electron-phonon interaction was accounted for by the two-temperature model, which relates $T_{e}$ and $T_{L}$ via:

$$C_{e}T_{e}\frac{\partial T_{e}}{\partial t}=\nabla\cdot\left(K_{e}\nabla T_{e}\right)-g\left(T_{e}-T_{L}\right)+\omega_{0}\mathrm{Im}\left(\varepsilon\right)\left\langle E\left(r,t\right)\cdot\left.E\left(r,t\right)\right\rangle\right.$$

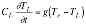
 (S4)

where
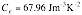
 is the heat captacity of electrons and
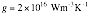
, which is a constant related to the coupling between electrons and phonons. Additionaly, $\left\langle E\left(r,t\right)\cdot\left.E\left(r,t\right)\right\rangle\right.$ is the time averaged electric field; $K_{e}=K_{e0}T_{e}$ is the electron heat diffusion constant with $K_{e0}=318\text{ Wm}^{\text{-1}}\text{K}^{\text{-1}}$ and $C_{L}$ is the lattice heat capacity.

Thus the two temperature model was solved, together with Maxwell’s equations, using COMSOL in order to simulate the pump probe experiment, with the results shown in Figures 4e-g.

**Supplementary Note 2.**

**SPP coupling efficiency definition**.

The numerically calculated SPP coupling efficiency ($CE$) is defined as:

$CE=P_{SPP}/P_{0}$ (S5)

where $P_{0}$ is the optical power incident on the slit aperture area and $P_{SPP}$ is the SPP power at the edge of the slit aperture. In order to accurately evaluate $P_{SPP}$, the SPP power $P_{x}$ is evaluated at a distance of$x=10$ μm from the edge of the slit, taking into account the propagation losses of the SPP mode:


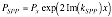
 (S6)

where $k_{SPP}=k_{0}\sqrt{\varepsilon_{d}\varepsilon_{m}/\left(\varepsilon_{d}+\varepsilon_{m}\right)}$ with $\varepsilon_{d}$ and $\varepsilon_{m}$ being the frequency-dependent dielectric and metal permittivities, respectively. Using these considerations, we were able to accurately determine a value for the coupling efficiency in the simulations.
